# Prevalence of CD8^+^ cytotoxic lymphocytes in human neoplasms

**DOI:** 10.1007/s13402-020-00496-7

**Published:** 2020-03-05

**Authors:** Niclas C. Blessin, Patrick Spriestersbach, Wenchao Li, Tim Mandelkow, David Dum, Ronald Simon, Claudia Hube-Magg, Florian Lutz, Florian Viehweger, Maximillian Lennartz, Christoph Fraune, Vera Nickelsen, Wilfried Fehrle, Cosima Göbel, Sören Weidemann, Till Clauditz, Patrick Lebok, Katharina Möller, Stefan Steurer, Jacob R. Izbicki, Guido Sauter, Sarah Minner, Frank Jacobsen, Andreas M. Luebke, Franziska Büscheck, Doris Höflmayer, Waldemar Wilczak, Eike Burandt, Andrea Hinsch

**Affiliations:** 1 Institute of Pathology, Martinistraße 52, University Medical Center Hamburg-Eppendorf, D-20246 Hamburg, Germany; 2 Department of General, Visceral and Thoracic Surgery, University Medical Center Hamburg-Eppendorf, Hamburg, Germany

**Keywords:** Tissue microarray, Immune checkpoint, Lymphocytic infiltrate, Cytotoxic T cells

## Abstract

**Purpose:**

Immune checkpoint inhibitors have recently been approved by the US FDA as first and/or second line therapy in a subset of cancer types. Recent evidence suggests that the quantity of tumor infiltrating lymphocytes (TILs) influences the likelihood of response to immune checkpoint inhibitors. Here, we set out to assess the density of CD8^+^ lymphocytes in a wide range of different cancer types and subtypes.

**Methods:**

The density of CD8^+^ lymphocytes was compared across different cancer types using tissue microarrays (TMAs) composed of up to 50 tumor samples each from 84 different cancer types and subtypes. In total 2652 cancers and 608 normal tissues were successfully analyzed by CD8 immunohistochemistry followed by automated image analysis of digitized slides.

**Results:**

We found that the median CD8^+^ lymphocyte counts ranged from 6 cells/mm^2^ in pleomorphic adenoma up to 1573 cells/mm^2^ in Hodgkin’s lymphoma. The CD8 counts were generally lower in normal tissues compared to cancer tissues. Blood vessels of the spleen were the only non-lymphatic tissue staining positive for CD8. Tumor types approved for checkpoint inhibitor therapy, including malignant melanoma (81), muscle invasive urothelial carcinoma (119), small cell lung cancer (120), clear cell renal cell cancer (153), squamous cell carcinoma (189) and adenocarcinoma of the lung (328) as well as Hodgkin’s lymphoma (1573) were all ranking among the upper half of our list. Comparably high CD8 densities (median cells/mm^2^) were also found in several rare and aggressive cancer types including Merkel cell carcinoma (70), angiosarcoma (95), anaplastic thyroid cancer (156) and embryonal carcinoma of the testis (186). In 73 of the 84 analyzed cancer types, the highly variable CD8 counts occasionally exceeded the average CD8 count of tumors for which checkpoint inhibitors have been approved.

**Conclusion:**

These data support the concept that among most tumor types at least some individual cancers may benefit from treatment with immune checkpoint inhibitors.

**Electronic supplementary material:**

The online version of this article (10.1007/s13402-020-00496-7) contains supplementary material, which is available to authorized users.

## Introduction

Cancer drugs targeting the host immune reaction are increasingly employed in cancer therapy. Immune checkpoint inhibitors such as Pembrolizumab, Nivolumab and Atzolizumab, directed against programmed death-1 (PD-1) or its ligand 1 (PD-L1), have recently been approved by the US FDA as first and/or second line therapy in various cancer types including melanoma, non-small cell lung cancer, small cell lung cancer, renal cell carcinoma, urothelial carcinoma, cervical cancer and Hodgkin’s lymphoma [1–8]. Several additional drugs targeting the PD-1/PD-L1 system and other immune checkpoints or their ligands are currently being investigated in clinical trials. It is expected that the number of approved immune checkpoint inhibitors and the cancer types for which they are being utilized will increase markedly in the coming years [9].

Despite striking successes of these therapies in many patients, as yet the majority of treated individuals only shows a partial response [10]. Predictive tests that identify suited patients are urgently needed. An increasing number of publications has demonstrated that the number of tumor infiltrating lymphocytes (TILs) is one relevant factor determining the potential response of a cancer to checkpoint inhibitors [11]. As these medications combat cancer by stimulating cytotoxic lymphocytes, it is thought that the presence of more lymphocytes may increase the likelihood for a successful application of immune checkpoint inhibitors. Even in absence of specific therapy, tumors with high lymphocyte content such as medullary breast cancers, seminomas or colorectal carcinomas are often characterized by a particularly good prognosis [11–13]. Many studies describing (TILs) have shown associations with tumor phenotype, patient outcome or response to therapy [14–16]. Altogether, these data indicate that the number of lymphocytes in cancer tissues is of clinical importance.

Given the growing interest in the lymphocyte content of tumors, we utilized tissue microarrays (TMAs) composed of up to 50 tumors each from 84 different cancer types and subtypes to compare the density of CD8^+^ lymphocytes. The data provide a systematic overview with respect to the degree of cytotoxic T cell involvement in different cancers.

## Material and methods

### Patients and tissues

**Fig. 1:**
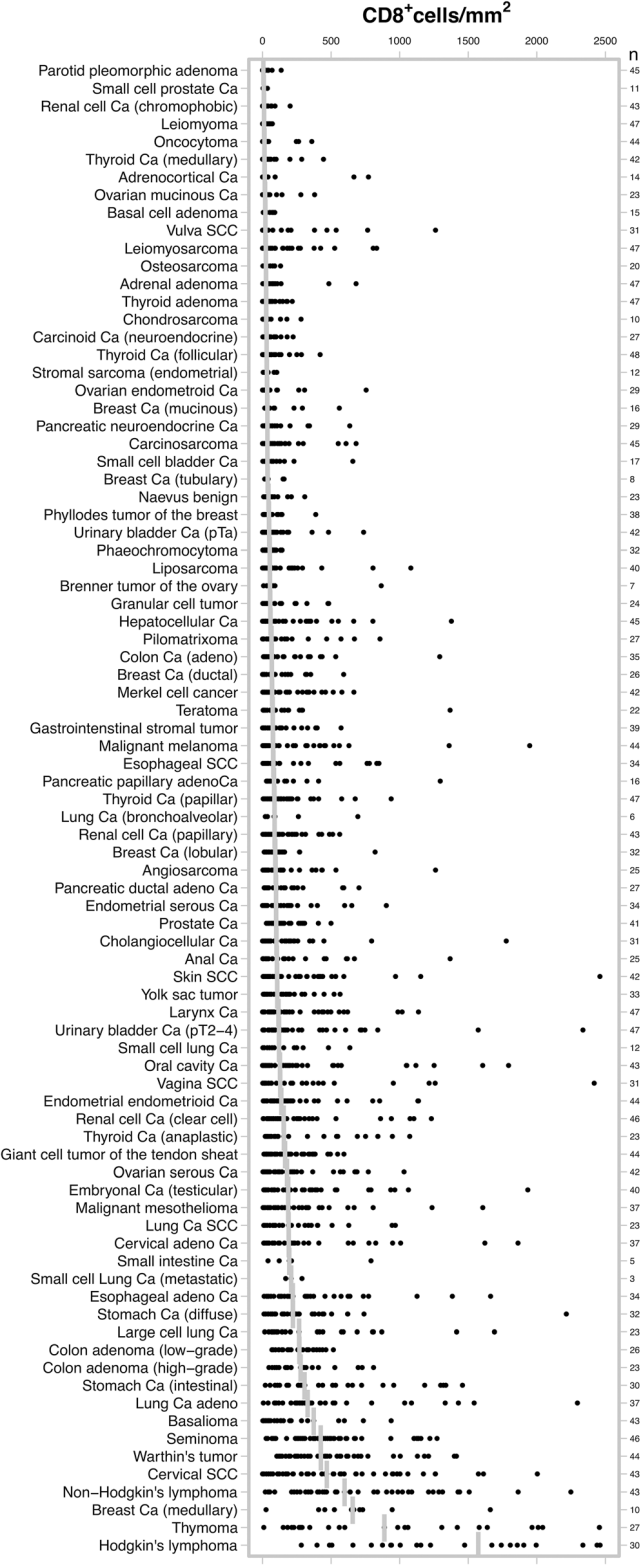
Distribution of CD8^+^ cell density (cell/mm^2^) across 84 different human tumor entities. In total 3339 tumor samples, represented by the black dots, were analyzed. The vertical bars indicate the median density per entity. Ca, carcinoma; SCC, squamous cell carcinoma

Formalin fixed paraffin embedded tissue samples from 3659 patients representing more than 80 different tumor types and subtypes and more than 70 different normal tissue types were retrieved from the archives of the Institute of Pathology at the University Medical Center Hamburg-Eppendorf. A pathologist identified representative cancerous and normal human tissue areas to assemble two different types of tissue microarrays (TMAs) from these samples: The first, a multitumor (TMA), contained 4–50 samples each from 84 different human tumor types and subtypes, as shown in Fig. 1. The samples of this first TMA were distributed among 8 different TMA blocks, each containing between 454 and 532 samples. The tissue cores were selected for a high tumor cell content on hematoxylin & eosin stained tissue sections of the donor tissue blocks, but not for particular features connected to the lymphocyte content such as the presence or density of infiltrating lymphocytes. The second TMA was composed of normal appearing tissues taken from organs removed for non-tumor reasons and encompassed 8 samples each of 76 different normal tissue types (608 samples on one slide), as shown in Supplementary Fig. 1. The exact composition of the study samples is shown in Table 1 and Supplementary Table 1. For both TMAs, a single 0.6 mm tissue punch was taken from each tissue sample. The TMA construction process was described previously [17]. The use of archived tissues has been approved by local laws (HmgKhG §12) and the local ethics committee (Ethics Commission Hamburg, WF-049/09). All studies have been carried out in compliance with the Helsinki Declaration.

**Table 1:** CD8^+^ cell densities (cells/mm^2^) in 84 different tumor types

Tumor entity	*N*	Mean	SD	Median	Max
Parotid pleomorphic adenoma	45	15	24	6	136
Small cell prostate Ca	11	14	10	9	35
Renal cell Ca (chromophobic)	43	23	35	10	201
Leiomyoma	47	19	18	11	71
Oncocytoma	44	33	72	13	359
Thyroid Ca (medullary)	42	43	83	14	445
Adrenocortical Ca	14	120	256	15	772
Ovarian mucinous Ca	23	53	95	18	380
Basal cell adenoma	15	31	28	20	89
Vulva SCC	31	141	279	21	1261
Leiomyosarcoma	47	113	193	24	832
Osteosarcoma	20	33	33	24	132
Adrenal adenoma	47	60	118	26	682
Thyroid adenoma	47	44	51	26	217
Chondrosarcoma	10	72	95	27	281
Carcinoid Ca (neuroendocrine)	27	53	55	30	223
Thyroid Ca (follicular)	48	58	80	30	420
Stromal sarcoma (endometrial)	12	43	35	31	104
Ovarian endometroid Ca	29	76	149	33	756
Breast Ca (mucinous)	16	98	146	35	560
Pancreatic neuroendocrine Ca	29	84	137	35	636
Carcinosarcoma	45	98	156	35	682
Small cell bladder Ca	17	94	159	36	658
Breast Ca (tubulary)	8	75	65	38	157
Naevus benign	23	69	72	43	308
Basalioma	42	142	212	46	938
Phyllodes tumor of the breast	38	62	67	46	388
Urinary bladder Ca (pTa)	42	89	138	48	737
Phaeochromocytoma	32	55	33	52	142
Liposarcoma	40	124	215	53	1081
Brenner tumor of the ovary	7	165	310	54	866
Granular cell tumor	24	118	138	55	483
Hepatocellular Ca	45	180	264	58	1377
Pilomatrixoma	27	157	227	65	856
Colon Ca (adeno)	35	169	243	67	1292
Breast Ca (ductal)	26	121	138	67	592
Merkel cell cancer	42	156	180	70	666
Teratoma	22	156	286	74	1368
Gastrointenstinal stromal tumor	39	115	137	75	573
Esophageal SCC	34	205	261	78	849
Malignant melanoma	43	231	371	81	1948
Pancreatic papillary adenoCa	16	199	313	83	1296
Thyroid Ca (papillar)	47	157	183	86	938
Lung Ca (bronchoalveolar)	6	198	258	87	695
Renal cell Ca (papillary)	43	141	149	88	563
Breast Ca (lobular)	32	119	143	92	821
Angiosarcoma	25	199	272	95	1262
Pancreatic ductal adeno Ca	27	158	188	98	703
Endometrial serous Ca	34	161	206	98	903
Prostate Ca	41	139	103	99	499
Cholangiocellular Ca	31	208	334	101	1777
Anal Ca	25	229	320	104	1368
Skin SCC	42	276	431	106	2460
Yolk sac tumor	33	145	154	107	566
Larynx Ca	47	230	281	115	1137
Urinary bladder Ca (pT2–4)	47	267	432	119	2336
Small cell lung Ca	12	192	200	120	637
Oral cavity Ca	43	304	452	127	1794
Vagina SCC	31	313	515	128	2418
Endometrial endometrioid Ca	44	237	283	137	1137
Renal cell Ca (clear cell)	46	306	479	153	2756
Thyroid Ca (anaplastic)	23	349	351	156	1073
Giant cell tumor of the tendon sheat	44	197	154	164	594
Ovarian serous Ca	42	235	248	178	1032
Embryonal Ca (testicular)	40	322	393	186	1934
Malignant mesothelioma	37	356	541	187	2844
Lung Ca SCC	23	274	273	189	969
Cervical adeno Ca	37	340	434	191	1863
Small intestine Ca	5	272	298	195	791
Small cell Lung Ca (metastatic)	3	222	61	208	288
Esophageal adeno Ca	34	378	386	220	1663
Stomach Ca (diffuse)	32	315	395	221	2215
Large cell lung Ca	23	440	446	266	1691
Colon adenoma (low-grade)	26	256	133	268	517
Colon adenoma (high-grade)	23	296	212	277	810
Stomach Ca (intestinal)	30	518	432	306	1458
Lung Ca adeno	37	488	488	328	2296
Seminoma	46	492	348	424	1272
Warthin’s tumor	44	501	356	425	1414
Cervical SCC	43	574	491	468	2004
Non-Hodgkin’s lymphoma	43	720	520	598	2248
Breast Ca (medullary)	10	677	424	657	1661
Thymoma	27	1017	833	889	2740
Hodgkin’s lymphoma	30	1649	954	1573	4100

### Immunohistochemistry

Freshly cut TMA sections were all stained for CD8 in one run on the same day. The slides were deparaffinized, rehydrated, exposed to heat-induced antigen retrieval for 15 min at 98 °C in pH 9 DAKO target retrieval Solution (S2367) using a DAKO PT-LINK device, and then transferred to a DAKO Link 48 autostainer device. The autostainer protocol includes peroxidase blocking for 5 min (DAKO, Envision Flex-Kit 8002) and subsequent incubations with the primary antibody (Oncodianova, mouse monoclonal antibody, Clone TC8, dilution 1:200) for 20 min at room temperature, Flex HRP (DAKO EnVision Flex-Kit 8002) for 20 min, DAB-Chromogen (DAKO EnVision Flex-Kit 8002) for 10 min, and a final incubation with Hämatoxylin (DAKO K8008) for 5 min.

### Definition of compartments and quantification of CD8 immunostaining

Digital images of stained slides were acquired using a Leica Aperio VERSA 8 automated microscope. TMA spots were automatically identified and analyzed using HALO™ (Indica Labs, US) in conjunction with the following procedure: Every TMA slide was scanned at 40 × magnification. Digital images were segmented using the HALO Tissue Microarray module to identify and annotate individual tissue spots. If necessary, the segmentation was corrected manually. The HALO Membrane IHC Quantification module was used to determine the number of CD8^+^ cells in each tissue spot and to measure the exact area of each tissue spot. The latter step was performed to compensate for uneven or incomplete tissue spots (average: 0.327 mm^2^). The number of stained cells and the area in square millimeters of each individual spot was used to calculate the density of stained cells/mm^2^ (number of cells per square mm). Examples of the image analysis procedure of tumor spots with a low, intermediate and high density of CD8^+^ lymphocytes are given in Supplementary Fig. 2.

### Statistics

JMP Pro 12 software package (SAS Institute Inc., NC, US) and R version 3.5.1 (The R foundation) [18, 19] were used to plot the data, to calculate the median values and to perform analysis of variance (ANOVA).

## Results

**Fig. 2:**
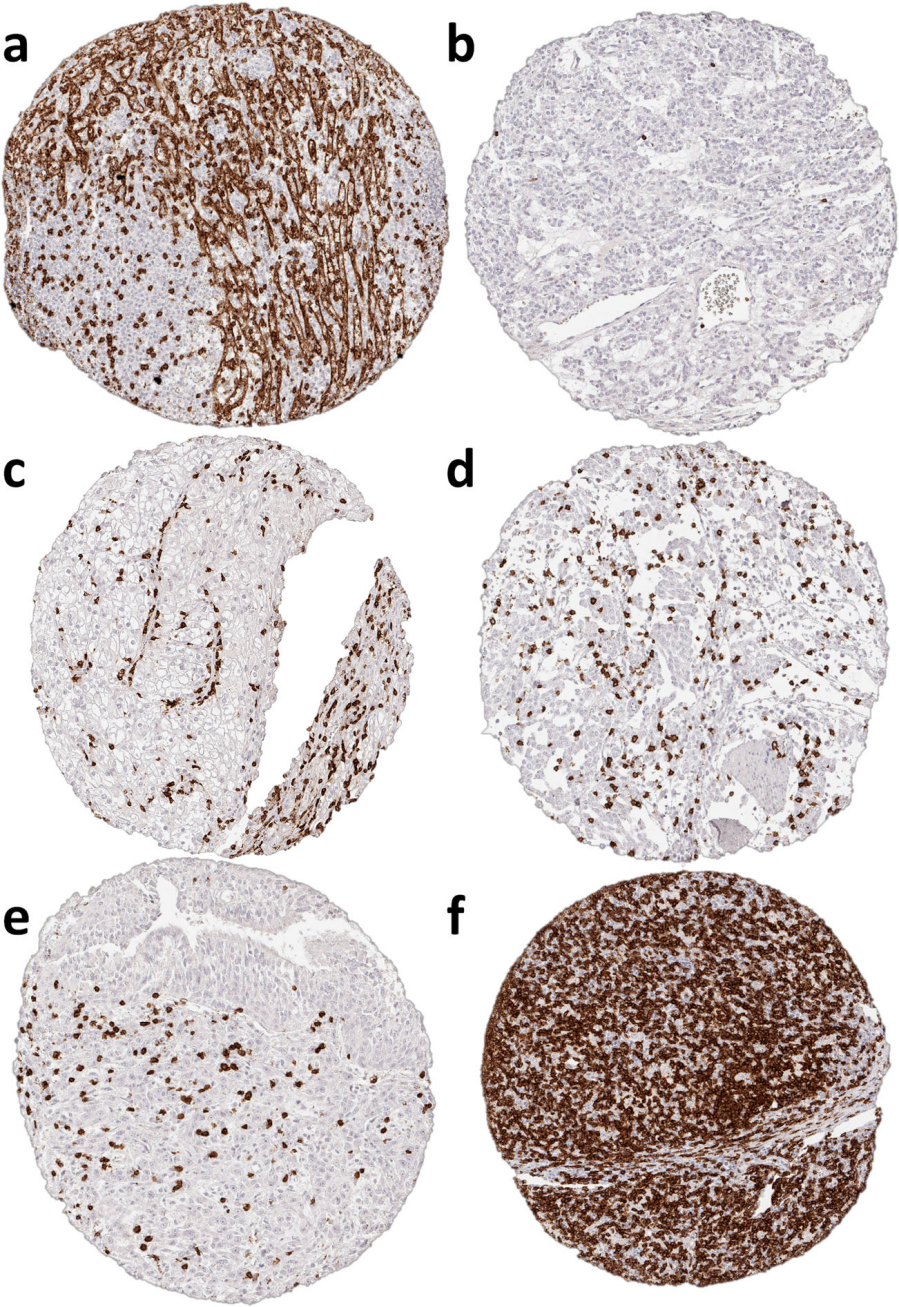
Representative pictures of CD8 immunohistochemistry in (**a**) healthy spleen, (**b**) pleomorphic adenoma, (**c**) clear cell renal cell cancer, (**d**) urinary bladder cancer, (**e**) squamous lung cancer and (**f**) Hodgkin’s lymphoma. Magnification 100×; TMA spot size 0.6 mm

A total of 2652 (72%) of 3659 tumor samples and 608 (100%) of 608 normal tissue samples were interpretable in our TMA analysis. The remaining 1007 spots were excluded due to missing tissue or the absence of unequivocal cancer cells in the TMA spot. The number of interpretable spots for each tumor type ranged from 3 to 48 samples (mean: 32 ± 13). The vast majority, i.e., 2602 (98%) of the 2652 tissue spots, contained CD8^+^ lymphocytes, although there were 50 (2%) samples that completely lacked CD8^+^ T cells. These were from 31 different tumor entities. All data are summarized in Table 1 and Fig. 1. The median number of CD8^+^ lymphocytes was highly variable and ranged from 6 cells/mm^2^ in pleomorphic adenoma (Fig. 2b) up to 1572 cells/mm^2^ in Hodgkin’s lymphoma (Fig. 2f). The top positions in our ranking order were held by tumor entities known for their high lymphocyte density such as seminoma (median: 424), Warthin’s tumor (median: 425), medullary breast cancer (median: 657), and thymoma (median: 889). Also, cancers in tissues with particularly high lymphocyte densities such as stomach cancer (median: 306) or cervical squamous cell carcinoma (median: 468) exhibited high CD8 values. At the bottom end of our list were pleomorphic adenoma of the parotis (median: 6), small cell cancer of prostate (median: 9), leiomyoma (median: 10) and oncocytoma (median: 13).

580 (95%) of the 608 tissue spots representing 58 (76%) of the 76 analyzed healthy normal tissues contained CD8^+^ lymphocytes. The median number of CD8^+^ lymphocytes was highly variable ranging from 0 cells/mm^2^ in the white matter of cerebrum up to 1643 cells/mm^2^ in the spleen. Blood vessels of the spleen were the only non-lymphatic tissue staining positive for CD8. The data are summarized in Supplementary Table 1 and Supplementary Fig. 1.

## Discussion

**Fig. 3:**
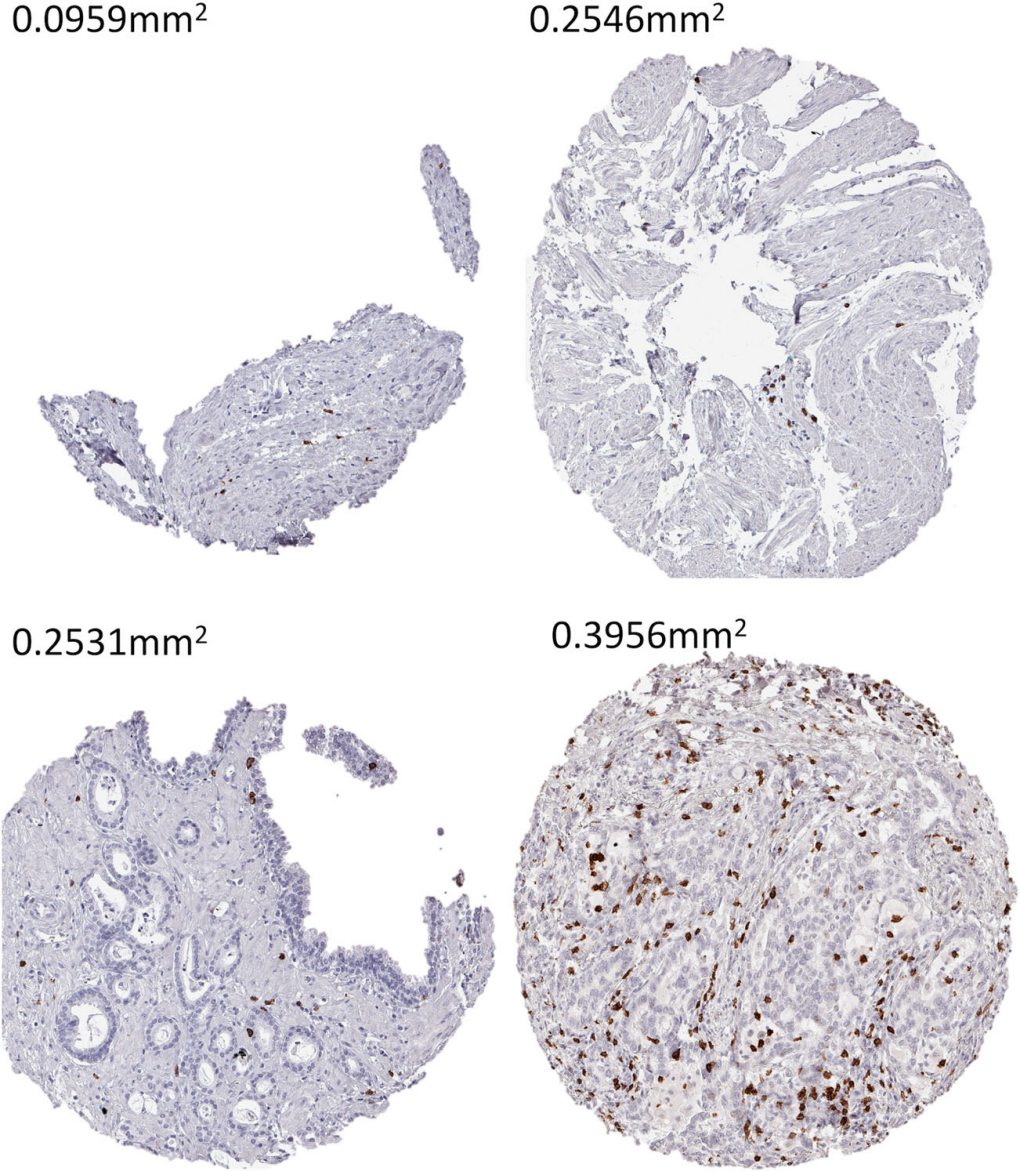
Area measurements of representative TMA cores that show an uneven or incomplete shape. Magnification 100×; TMA spot size 0.6 mm**.** Note that the tissue spots may have a larger area as theoretically expected from a 0.6 mm tissue punch (0.283 mm^2^). This is because the tissue expands when mounted on a slide

The data from this study provide a comprehensive overview of the density of tumor infiltrating CD8^+^ lymphocytes across a large number of different human tumor types. Numerous studies have recently quantified immune cell infiltrations in and around tumors. Most of these have used semi-quantitative methods to describe their data such as cell counts per high-power field or per predefined tissue area, positive pixel counts per area, semi-quantitative estimation of TIL numbers or the fraction of TILs in relation to the total intra-tumoral stroma [20–26]. Data on lymphocytic infiltration in cancers cannot always easily be compared between studies because objective metric data are often lacking. In this project, we described all data as “cells/mm^2^” in order to generate as comparable data as possible. An automated approach was developed that eliminates differences in tissue quantity occurring in TMA studies. Most spots are typically intact in TMA studies, but some are incomplete (Fig. 3). This may be due to specific properties of the arrayed tissue or may be caused by technical issues during embedding of tissue cores in a TMA or during sectioning. The validity of our approach is supported by our finding that tumor types among the top positions in our ranking order are morphologically characterized by high lymphocyte densities, such as seminoma, Warthin’s tumor, medullary breast cancer or thymoma. Other cancers with particularly high lymphocyte densities are those occurring in areas with a high density of mucosal lymphocytes, such as cervical or stomach cancer.

Several earlier CD8 density studies in cancers employing automated image analysis or manual cell counting to quantitate TILs in terms of “cells per mm^2^” yielded results that were comparable to those from our study. For example, an assessment of 220 gastric cancers by automated image analysis showed a median count of 436 CD8^+^ cells/mm^2^, which fits well with our results (mean: 413 CD8^+^ cells/mm^2^ in 62 intestinal and diffuse stomach cancers) [27]. Another study that counted T cells manually in 102 cervical squamous cell cancers reported a median of 441 CD8^+^ T cells/mm^2^, which is very similar to the median value of 468 CD8^+^ T cells/mm^2^ in our 43 patients [20]. One earlier study by Steele et al. [28] has used “cells/mm^2^” to compare the density of CD8^+^ cells in 8 different cancer types in a study analyzing the largest possible area per cancer on large sections. Several relevant observations were comparable between our and their study (Table 2), such as relatively low densities of CD8^+^ cells in kidney and prostate cancers and a high density in non-small cell lung cancer. The largest discrepancy was seen in pancreatic cancers, in which Steele at al. found 316 cells/mm^2^ and we found 93 cells/mm^2^ on average. It is a peculiarity of pancreatic cancer that it contains a high amount of tumor stroma and that the lymphocytes mostly reside in the stroma. It cannot be excluded that the selection process of tissues for TMA manufacturing has resulted in an overrepresentation of cancer cell-rich tissues with less stroma, as these are easier to target during the array manufacturing process.

**Table 2:** Comparison of median CD8^+^ cell densities with findings by Steele et al.

	Current Study			Steele et al.	
Tumor entity	Subgroup	*n*	Density (cells/mm^2^)	*n*	Density (cells/mm^2^)
Bladder	Total	89	76	50	124
	pTa	42	48		
	pT2–4	47	119		
Pancreatic	Total	43	93	48	316
	Papillary	16	83		
	Ductal adenocarcinoma	27	98		
Prostate	Total	41	99	50	143
RCC	Total	89	112	49	104
	Papillary	43	88		
	Clear cell	46	153		
Head and neck	Total	90	118	50	220
	Larynx	47	115		
	Oral cavity	43	127		
Lung squamous	Total	23	189	41	346
Gastroesophageal	Total	153	221	50	207
	Esophageal SCC	34	78		
	Esophageal AdenoCa	34	220		
	Stomach Ca (diffus)	32	221		
	Stomach Ca (intestinal)	30	306		
Lung non-squamous	Total	60	328	42	238
	Large cell	23	266		
	Adenocarcinoma	37	328		

Cancer types for which treatment regimens with immune checkpoint inhibitors are already approved, such as malignant melanoma, muscle invasive urothelial carcinoma, clear cell renal cell cancer, squamous cell cancer and adenocarcinoma of the lung, small cell lung cancer and Hodgkin’s lymphoma, all ranked in the upper half of our list. This supports the concept that a high number of tumor infiltrating lymphocytes goes along with an increased likelihood for response to immune checkpoint inhibitors. The median content of CD8^+^ cells was 248 cells/mm^2^ for these tumor types. That 73 of our 84 analyzed tumor types had - at least occasionally - CD8^+^ cell counts that were higher than 248 cells/mm^2^ may be viewed as an argument for the applicability of immune checkpoint inhibitors - and probably other forms of immunotherapy - to a wide variety of different types of cancer. However, the CD8-positive cell density was found to be highly variable in most analyzed cancer types. All analyzed cancer types had at least one tumor with a CD8^+^ cell count below 282/mm^2^. This is in line with clinical observations that only a fraction of individual cancers from a certain tumor type will eventually respond favorably to treatment [29].

We identified several rare and aggressive tumor types that frequently exhibited a high density of CD8^+^ cells. These included anaplastic thyroid cancer, anal carcinoma, embryonal carcinoma of the testis, Merkel cell carcinoma, angiosarcoma and squamous cell carcinoma of the vagina. These cancers are candidates for a thorough assessment of the efficacy of immune checkpoint inhibitors. A clinically relevant effect of the lymphocyte content has already been suggested for some of these tumors, including reports on successful therapy attempts with checkpoint inhibitors. For Merkel cell carcinoma, a prognostic role of the associated immune cell count has been described in several studies [30, 31]. Furthermore, an objective response rate of more than 50% in a cohort of 26 Merkel cell carcinoma patients treated with anti-PD-1 therapy [32] has led to several ongoing phase I (NCT02488759, NCT02584829), II (NCT02196961) and III (NCT03271372) studies for this tumor entity. Also a patient with anaplastic thyroid cancer has recently reached complete radiographic and clinical remission after anti-PD-1 therapy [33]. Combination therapies can also be efficient in these tumor types as demonstrated by the success of combining monoclonal antibodies targeting PD-1 and CTLA-4 in angio- and other sarcoma types [34]. Moreover, a prognostic role of the CD8^+^ cell count was recently suggested in angiosarcoma [35].

**Fig. 4:**
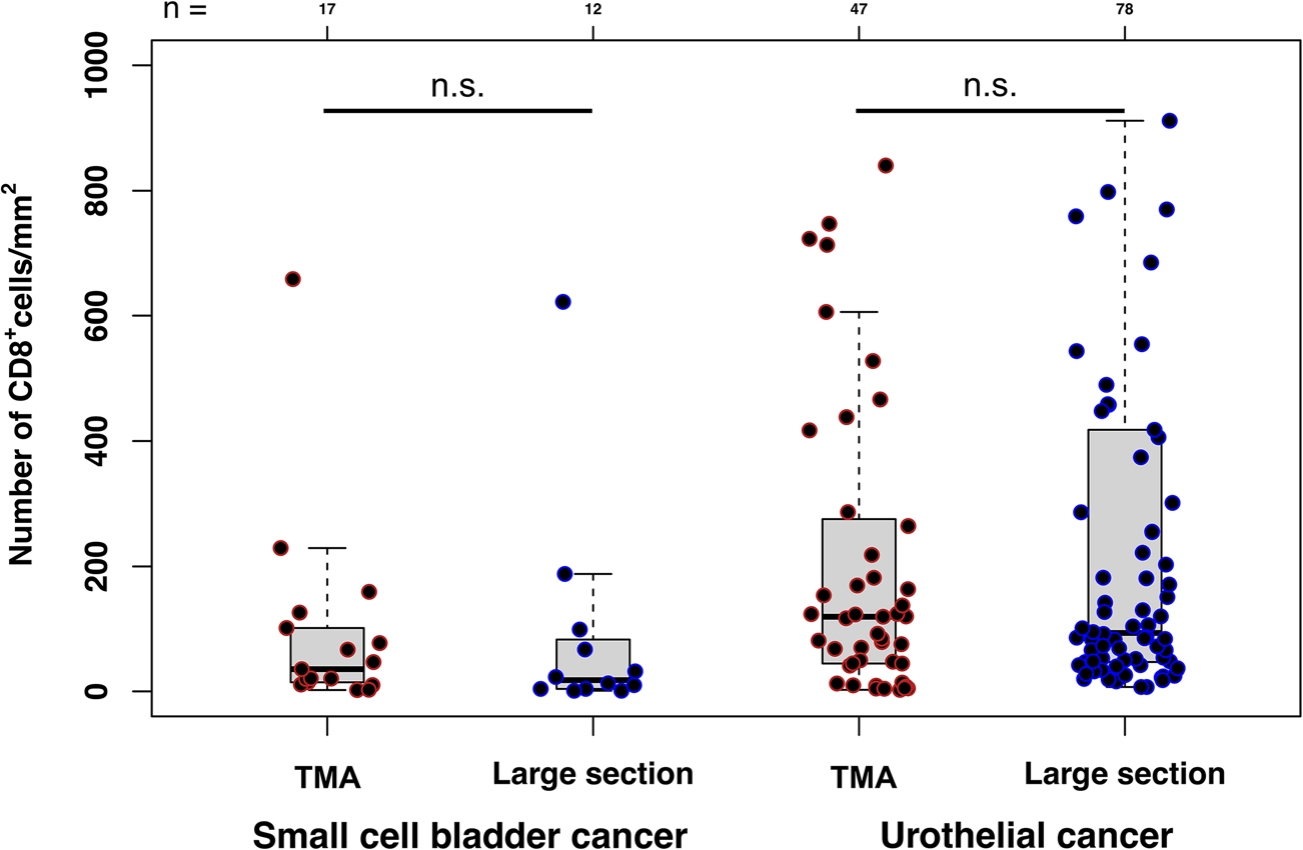
Comparison of CD8^+^ cell densities in large section and TMA formats. TMA, multi-tumor tissue microarray

The premise that the number of lymphocytes in a cancer is linked to the likelihood of a positive response to immune checkpoint inhibitors is widely accepted [36]. Many studies have separately assessed lymphocyte counts in the tumor center and in its periphery [13, 37–39]. Some studies have recently suggested that the lymphocyte content at the tumor periphery may be more important clinically than the lymphocyte content in the tumor center. For example, in a study on 46 patients with metastatic melanoma, Tumeh et al. found that response to anti-PD-1 therapy was associated with higher CD8^+^ cell densities within the invasive margin [37]. In another study on metastatic colorectal cancer, the density of immune cells at the invasive margin predicted response to conventional chemotherapy [40]. However, quantification of TILs at the tumor margin is hampered by a “non-standardized” structure of the tumor periphery. For several tumor types such as bladder cancer, colorectal cancer and stomach cancer, different growth patterns have been described [41–43]. TIL quantification is easy in tumors with a solid growth and a clearly defined tumor margin, but is much more difficult in cancers showing insular or diffuse growth patterns with irregular borders. In contrast, lymphocyte quantification in the tumor center is independent of tumor growth patterns and thus simpler and perhaps more reproducible to measure than in the periphery. The high concordance between large sections and TMA CD8 counts in 17 small cell bladder carcinomas and in 47 urothelial cancers demonstrates that representative lymphocyte quantification is possible for tumor centers on a 0.6 mm tissue core (Fig. 4).

Tissue microarrays with small 0.6 mm tissue cores were used in this study. When we introduced the TMA technology in 1998, several authors criticized the small diameter of the TMA spots. However, almost 20 years later, every known association between immune cell quantification and patient outcome [13, 44, 45] or between molecular markers and clinico-pathological features [46–49] have been successfully reproduced in TMA studies. In an earlier study on p53 staining, we even showed that the 0.6 mm TMA format provided better predictive power compared to the large section format technology [46]. Contrary to initial expectations, tissue heterogeneity does not negatively influence the ability of TMA spots to detect associations between molecular markers and tumor features. This is because of the high number of samples that are typically used in such analyses, as well as the high degree of standardization of the experimental conditions that cannot be achieved in standard slide-by-slide analyses [50–53].

Our analysis also included 76 different types of normal tissues, which apart from lymphatic tissues, exhibited much lower CD8^+^ lymphocyte counts than the tumors. Spleen blood vessels were the only non-lymphoid cell type staining unequivocally positive for CD8. CD8 expression in splenic vessels was first described by Buckley et al. in 1985 [54]. Further studies revealed that only the alpha chain of the CD8 molecule is expressed by the venous sinus-lining cells in the spleen’s red pulp (also known as littoral cells) and that this expression is unique to human spleen [55].

In summary, the data presented in this study provide a comprehensive overview of CD8 positivity across human normal tissues and cancers. Cancer types with high densities of CD8^+^ immune cells may be the best candidates for studies assessing the efficacy of immune checkpoint therapies.

## Electronic supplementary material

ESM 1 (DOCX 136066 kb)
