# Supplementary material for: Prevalence of CD8+ cytotoxic lymphocytes in human neoplasms
Source: Cell Oncol (Dordr). 2020 Mar 5;43(3):421–30. doi: 10.1007/s13402-020-00496-7 (PMC7214387; doi:10.1007/s13402-020-00496-7)
Supplement: Supplementary file 1 — (DOCX 136066 kb) [file 13402_2020_496_MOESM1_ESM.docx]

**Cellular Oncology**

**Distribution of CD8^+^ cytotoxic lymphocytes in human neoplasms**

Niclas C. Blessin^1^*, Patrick Spriestersbach^1^*, Wenchao Li^1^, Tim Mandelkow^1^, David Dum^1^, Ronald Simon^#1^, Claudia Hube-Magg^1^, Florian Lutz^1^, Florian Viehweger^1^, Maximillian Lennartz^1^, Christoph Fraune^1^, Vera Nickelsen^1^, Wilfried Fehrle^1^, Cosima Göbel^1^, Sören Weidemann^1^, Till Clauditz^1^, Patrick Lebok^1^, Katharina Möller^1^, Stefan Steurer^1^, Jacob R. Izbicki^2^, Guido Sauter^1^, Sarah Minner^1^, Frank Jacobsen^1^, Andreas M. Luebke^1^, Franziska Büscheck^1^, Doris Höflmayer^1^, Waldemar Wilczak^1^, Eike Burandt^1^, Andrea Hinsch^1^

^1^Department of Pathology, University Medical Center Hamburg-Eppendorf, Germany

^2^Department of General, Visceral and Thoracic Surgery, University Medical Center Hamburg-Eppendorf, Germany

*** These authors have contributed equally to the study**

\# Corresponding author: Dr. Ronald Simon, Institute of Pathology, Martinistraße 52, University Medical Center Hamburg-Eppendorf, D-20246 Hamburg, Germany, e-mail: [r.simon@uke.de](mailto:r.simon@uke.de), phone +49 (0)40 7410-5-7214, ORCID: 0000-0003-0158-4258

**Supplementary information**

| **Supplementary Table 1**. CD8^+^ cell densities in 76 different normal tissues. | | | | | | |
| --- | --- | --- | --- | --- | --- | --- |
| **Tissue** | **N** | **Mean** | **SD** | **Median** | **Max** |  |
|  | | | | | | |
| Cerebrum white | 8 | 3 | 6 | 0 | 18 |  |
| Striated muscle | 8 | 34 | 82 | 2 | 237 |  |
| Cerebrum grey | 8 | 2 | 2 | 2 | 5 |  |
| Cerebellum cortex (Stratum moleculare) | 8 | 5 | 4 | 3 | 12 |  |
| Aorta media | 8 | 18 | 29 | 5 | 87 |  |
| Cerebellum grey (Stratum neuronorum) | 8 | 13 | 24 | 6 | 72 |  |
| Testis | 8 | 11 | 14 | 6 | 46 |  |
| Urinary bladder muscular wall | 8 | 15 | 18 | 7 | 57 |  |
| Heart | 8 | 11 | 11 | 8 | 33 |  |
| Tongue muscle | 8 | 11 | 9 | 9 | 33 |  |
| Kidney medulla | 8 | 66 | 101 | 10 | 254 |  |
| Ovary corpus luteum | 8 | 26 | 42 | 12 | 127 |  |
| Esophagus muscular wall | 8 | 19 | 12 | 15 | 41 |  |
| Colon descendens muscular wall | 8 | 22 | 22 | 16 | 75 |  |
| Stomach muscular wall | 8 | 13 | 7 | 16 | 19 |  |
| Aorta endothelium | 8 | 21 | 19 | 18 | 52 |  |
| Penis corpos spongiosum | 8 | 19 | 9 | 18 | 33 |  |
| Kidney cortex | 8 | 36 | 33 | 21 | 111 |  |
| Ovary stroma | 8 | 33 | 22 | 30 | 80 |  |
| Pituitary anterior lobe | 8 | 37 | 20 | 31 | 75 |  |
| Fat | 8 | 33 | 27 | 32 | 88 |  |
| Appendix muscular wall | 8 | 47 | 37 | 32 | 129 |  |
| Tyroid gland | 8 | 40 | 38 | 32 | 117 |  |
| Pituitary posterior lobe/infundibulum | 8 | 76 | 93 | 34 | 284 |  |
| Kidney pelvis muscular wall | 8 | 76 | 140 | 34 | 421 |  |
| Uterus myometrium | 8 | 58 | 57 | 34 | 166 |  |
| Epididymis | 8 | 49 | 35 | 36 | 133 |  |
| Ovary follicular cyst | 8 | 40 | 28 | 37 | 101 |  |
| Parathyroid | 8 | 93 | 133 | 37 | 391 |  |
| Skin | 8 | 55 | 52 | 38 | 134 |  |
| Ileum muscularis | 8 | 50 | 37 | 41 | 120 |  |
| Adrenal gland | 8 | 70 | 59 | 45 | 199 |  |
| Pancreas | 8 | 47 | 24 | 46 | 87 |  |
| Seminal vesicle | 8 | 58 | 43 | 50 | 148 |  |
| Endocervix | 8 | 63 | 30 | 57 | 125 |  |
| Breast | 8 | 96 | 97 | 62 | 329 |  |
| Parotid gland | 8 | 89 | 68 | 62 | 237 |  |
| Placenta early decidua | 8 | 102 | 89 | 70 | 275 |  |
| Kidney pelvis urothelium | 8 | 76 | 64 | 72 | 194 |  |
| Anal canal skin | 8 | 68 | 36 | 72 | 128 |  |
| Placenta early | 8 | 87 | 52 | 88 | 166 |  |
| Endometrium secretion | 8 | 96 | 45 | 100 | 167 |  |
| Submandibular gland | 8 | 135 | 116 | 104 | 345 |  |
| Skin hairfollicel and sebaceous glands | 8 | 145 | 120 | 106 | 425 |  |
| Liver | 8 | 142 | 126 | 107 | 422 |  |
| Bone marrow | 8 | 102 | 31 | 107 | 140 |  |
| Prostate | 8 | 108 | 67 | 122 | 180 |  |
| Anal canal transitional mucosa | 8 | 187 | 244 | 123 | 734 |  |
| Endometrium proliferation | 8 | 162 | 155 | 124 | 522 |  |
| Colon descendens mucosa | 8 | 215 | 234 | 133 | 556 |  |
| Esophagus squamous epithelium | 8 | 742 | 1305 | 133 | 3721 |  |
| Lip oral mucosa | 8 | 124 | 98 | 137 | 298 |  |
| Duodenum Brunner gland | 8 | 275 | 284 | 146 | 906 |  |
| Urinary bladder urothelium | 8 | 177 | 138 | 153 | 397 |  |
| Sublingual gland | 8 | 177 | 162 | 155 | 442 |  |
| Ektocervix | 8 | 126 | 85 | 156 | 245 |  |
| Placenta mature amnion and chorion | 8 | 186 | 162 | 177 | 430 |  |
| Bronchus glands | 8 | 222 | 167 | 203 | 579 |  |
| Rectum mucosa | 8 | 247 | 155 | 208 | 532 |  |
| Placenta mature | 8 | 259 | 154 | 212 | 613 |  |
| Gallbladder epithelium | 8 | 369 | 369 | 224 | 1053 |  |
| Sinus paranasales | 8 | 236 | 177 | 224 | 482 |  |
| Lung | 8 | 270 | 137 | 226 | 507 |  |
| Oral cavity | 8 | 244 | 193 | 228 | 490 |  |
| Stomach antrum | 8 | 333 | 287 | 280 | 861 |  |
| Duodenum mucosa | 8 | 403 | 367 | 290 | 1149 |  |
| Fallopian tube mucosa | 8 | 431 | 446 | 290 | 1493 |  |
| Bronchus mucosa | 8 | 343 | 308 | 313 | 1017 |  |
| Stomach corpus | 8 | 432 | 218 | 347 | 761 |  |
| Thymus | 8 | 505 | 343 | 427 | 1235 |  |
| Ileum mucosa | 8 | 487 | 346 | 454 | 1129 |  |
| Appendix mucosa | 8 | 634 | 429 | 628 | 1375 |  |
| Tonsil | 8 | 1113 | 825 | 921 | 2739 |  |
| Tonsil surface epithelium | 8 | 863 | 389 | 1001 | 1274 |  |
| Lymph node | 8 | 1587 | 628 | 1303 | 2689 |  |
| Spleen | 8 | 1674 | 514 | 1643 | 2453 |  |
|  | | | | | | |

**Supplementary Figure 1**. Distribution of CD8^+^ cell density (cell/mm2) across 76 different healthy human tissues. In total, 608 tissue cores, represented by the black dots, have been analyzed. The horizontal bars indicate the median density per normal tissue.

**Supplementary Figure 2 (next page).** Schematic representation of the workflow to detect and count CD8+ cells and to determine the area of the tissue spot. Three examples are given for tumors with low (top, colon cancer), intermediate (middle, head & neck squamous cell cancer) and high (bottom, Hodgkin’s lymphoma) CD8+ cell density. Note that tissue spots can have a higher area as theoretically expected from a 0.6mm tissue punch (0.283mm^2^). This is because the tissue expands when mounted on a slide.
